# Brain Region-Dependent Effects of Neuropeptide Y on Conditioned Social Fear and Anxiety-Like Behavior in Male Mice

**DOI:** 10.3390/ijms22073695

**Published:** 2021-04-02

**Authors:** Johannes Kornhuber, Iulia Zoicas

**Affiliations:** Department of Psychiatry and Psychotherapy, Friedrich-Alexander University Erlangen-Nürnberg (FAU), 91054 Erlangen, Germany; Johannes.Kornhuber@uk-erlangen.de

**Keywords:** social fear, conditioned fear, SFC, fear expression, anxiety, elevated plus-maze, neuropeptide Y, dorsolateral septum, central amygdala

## Abstract

Neuropeptide Y (NPY) has anxiolytic-like effects and facilitates the extinction of cued and contextual fear in rodents. We have previously shown that the intracerebroventricular administration of NPY reduces the expression of social fear in a mouse model of social fear conditioning (SFC). In the present study, we aimed to identify the brain regions that mediate these effects of NPY. We show that NPY (0.1 nmol/0.2 µL/side) reduces the expression of SFC-induced social fear in a brain-region-dependent manner. In more detail, NPY reduced the expression of social fear when administered into the dorsolateral septum (DLS) and central amygdala (CeA), but not when administered into the dorsal hippocampus (DH), medial amygdala (MeA) and basolateral amygdala (BLA). We also investigated whether the reduced expression of social fear might partly be due to a reduced anxiety-like behavior, and showed that NPY exerted anxiolytic-like effects when administered into the DH, DLS, CeA and BLA, but not when administered into the MeA. This study identifies the DLS and the CeA as brain regions mediating the effects of NPY on the expression of social fear and suggests that partly distinct neural circuitries mediate the effects of NPY on the expression of social fear and on anxiety-like behavior.

## 1. Introduction

Neuropeptide Y (NPY) is the most abundant and widely distributed neuropeptide in the mammalian brain. It regulates important biological and pathophysiological functions, such as blood pressure, food intake, neuroendocrine secretions, seizures, neuronal excitability and neuroplasticity [1,2,3,4,5,6]. These effects are mediated by at least five different G-protein-coupled receptors, among which the Y1, Y2, Y4 and Y5 subtypes are localized in the brain [7,8,9]. NPY is expressed at high levels in brain regions involved in emotional behavior and fear-related behavior, suggesting a regulatory role of NPY in these behaviors. The brain regions expressing NPY include, among others, the amygdala, hippocampus, septum, cerebral cortex, locus coeruleus, periaqueductal gray, basal ganglia, hypothalamus and thalamus [10,11,12]. Indeed, a considerable amount of the literature supports an anxiolytic function of NPY [13,14,15]. As such, intracerebroventricular (i.c.v.) administration of NPY has anxiolytic-like effects [16], which can also be seen after NPY administration directly into the central amygdala (CeA) [17], locus coeruleus [18], lateral septum [19], dentate gyrus and the CA1 region of the hippocampus [20], indicating that these brain regions mediate the anxiolytic-like effects of NPY. The anxiolytic-like effects can also be seen in rats with a higher innate number of NPY-positive cells in the CeA [21] and after localized overexpression of NPY within the amygdala [22]. Similar brain region-specific effects of NPY were described on social interaction. As such, NPY promotes social interaction when administered into the dorsolateral septum (DLS) [23] and basolateral amygdala (BLA) [24], but does not affect social interaction when administered into the intramedial septum [23] and CeA [24]. These anxiolytic and prosocial effects of NPY suggest its potential benefit in disorders associated with social anxiety and fear. This might also be suggested by the fact that NPY affects different aspects of fear-related behavior. In fear conditioning paradigms, for example, i.c.v.-administered NPY impairs the acquisition of cued and contextual fear [25,26,27], impairs the retention and retrieval of cued fear [28,29,30] and facilitates the extinction of cued and contextual fear [27,28,31,32,33]. The effects of NPY on the expression of cued fear also seem to be brain region-dependent, since NPY inhibits the expression of cued fear when administered into the BLA, but not when administered into the medial amygdala (MeA) [28,29]. These studies suggest that NPY acts as a resilience factor against exaggerated fear responses after stressful and adverse events and that these effects are brain-region-dependent. However, little is known about the involvement of NPY in the modulation of fear responses after adverse social events or about the neural circuitries mediating these effects.

Recently, in a model of social fear conditioning (SFC), we could show that i.c.v.-administered NPY reduces the expression of social fear, but does not alter the acquisition of social fear [34]. This suggests that although NPY does not prevent the formation of aversive social memories, it might improve the recovery from a traumatic social experience. Moreover, i.c.v.-administered NPY also reduces the expression of antidepressant-resistant social fear [35], further supporting its potential benefit in disorders associated with social anxiety and fear. In the present study, we aimed to identify the brain regions mediating the effects of NPY on the expression of social fear. Therefore, we administered NPY into the dorsal hippocampus (DH), DLS, CeA, MeA and BLA before social fear extinction. These brain regions were selected because the amygdala and the septo-hippocampal circuits have strong NPY-ergic innervation [10,11,36], and are important components of the neural circuitry controlling anxiety-related behaviors, fear-related behaviors, social behaviors and stress responses [36,37,38,39]. We also investigated whether the effects of NPY on the expression of social fear might be due to altered anxiety-like behavior.

## 2. Results

To investigate whether NPY alters the expression of social fear when administered into the DH, DLS, CeA, MeA and/or BLA, separate groups of conditioned (SFC^+^) and unconditioned (SFC^−^) mice were administered either vehicle (Veh; 0.2 µL/side) or NPY (0.1 nmol/0.2 µL/side) into these brain regions 10 min before social fear extinction on day 2. On day 1, during SFC, SFC^+^ mice received mild electric foot shocks each time they investigated an unknown conspecific, whereas SFC^−^ mice investigated an unknown conspecific without receiving foot shocks. On day 2, during social fear extinction, we assessed the time that the SFC^+^ and SFC^−^ mice spent investigating three empty cages (i.e., non-social investigation) and six unknown conspecifics enclosed in wire mesh cages (i.e., social investigation) as a read-out of non-social and social fear, respectively.

### 2.1. NPY Does Not Affect the Expression of Social Fear When Administered into the DH

**Figure 1:**
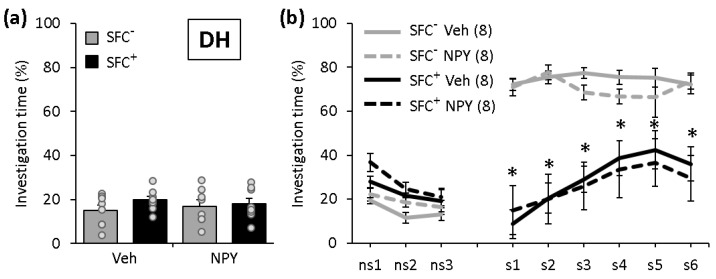
Neuropeptide Y (NPY) does not affect the expression of social fear when administered into the dorsal hippocampus (DH). (**a**) Pre-conditioned investigation of the non-social stimulus (empty cage) during social fear conditioning (SFC) on day 1. (**b**) Investigation of the non-social (ns1–ns3) and social (cages with mice; s1–s6) stimuli during social fear extinction on day 2. Unconditioned (SFC^−^) and conditioned (SFC^+^) mice were administered either vehicle (Veh; 0.2 µL/side) or NPY (0.1 nmol/0.2 µL/side) 10 min before social fear extinction. Data represent means ± SEM and numbers in parentheses indicate group sizes. *p* < 0.05 * vs. respective SFC^−^ controls.

During SFC on day 1, SFC^+^ and SFC^−^ mice showed similar investigation of the non-social stimulus (empty cage), reflecting a similar pre-conditioning non-social anxiety (Figure 1a; F(3,28) = 0.684; *p* = 0.570). All SFC^+^ mice received a similar number of foot shocks during SFC, reflecting similar levels of distress during conditioning and similar social fear learning (T(14) = −0.798; *p* = 0.438). During social fear extinction on day 2, all SFC^+^ and SFC^−^ mice showed a similar investigation of the non-social stimuli (three empty cages; ns1–ns3), indicating that SFC did not induce an unspecific non-social fear (Figure 1b). However, all SFC^+^ mice showed a reduced investigation of the social stimuli (six unknown conspecifics; s1–s6) compared with respective SFC^−^ mice, independent of treatment, reflecting an increased social fear (conditioning effect F(1,28) = 53.556; *p* < 0.001; conditioning x treatment effect F(1,28) = 0.024; *p* = 0.879). This indicates that NPY does not reduce the expression of social fear when administered into the DH.

### 2.2. NPY Reduces the Expression of Social Fear When Administered into the DLS

**Figure 2:**
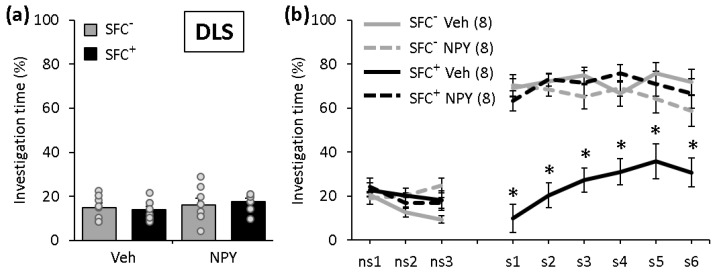
NPY reduces the expression of social fear when administered into the dorsolateral septum (DLS). (**a**) Pre-conditioned investigation of the non-social stimulus (empty cage) during SFC on day 1. (**b**) Investigation of the non-social (ns1–ns3) and social (cages with mice; s1–s6) stimuli during social fear extinction on day 2. Unconditioned (SFC^−^) and conditioned (SFC^+^) mice were administered either vehicle (Veh; 0.2 µL/side) or NPY (0.1 nmol/0.2 µL/side) 10 min before social fear extinction. Data represent means ± SEM and numbers in parentheses indicate group sizes. *p* < 0.05 * vs. all groups.

During SFC on day 1, SFC^+^ and SFC^−^ mice showed similar investigation of the non-social stimulus, reflecting a similar pre-conditioning non-social anxiety (Figure 2a; F(3,28) = 0.661; *p* = 0.583). All SFC^+^ mice received a similar number of foot shocks during SFC, reflecting similar levels of distress during conditioning and similar social fear learning (T(14) = 0.532; *p* = 0.603). During social fear extinction on day 2 (Figure 2b), all SFC^+^ and SFC^−^ mice showed a similar non-social investigation, indicating that SFC did not induce an unspecific non-social fear. While Veh-treated SFC^+^ mice showed a reduced social investigation compared with all other groups, reflecting social fear, NPY increased social investigation starting from the first social stimulus, reflecting a reduced expression of social fear (conditioning x treatment effect F(1,28) = 47.150; *p* < 0.001; stimulus x conditioning x treatment effect F(8,224) = 11.611; *p* < 0.001). This indicates that NPY reduces the expression of social fear when administered into the DLS.

### 2.3. NPY Reduces the Expression of Social Fear When Administered into the CeA, But Not When Administered into the MeA or BLA

**Figure 3:**
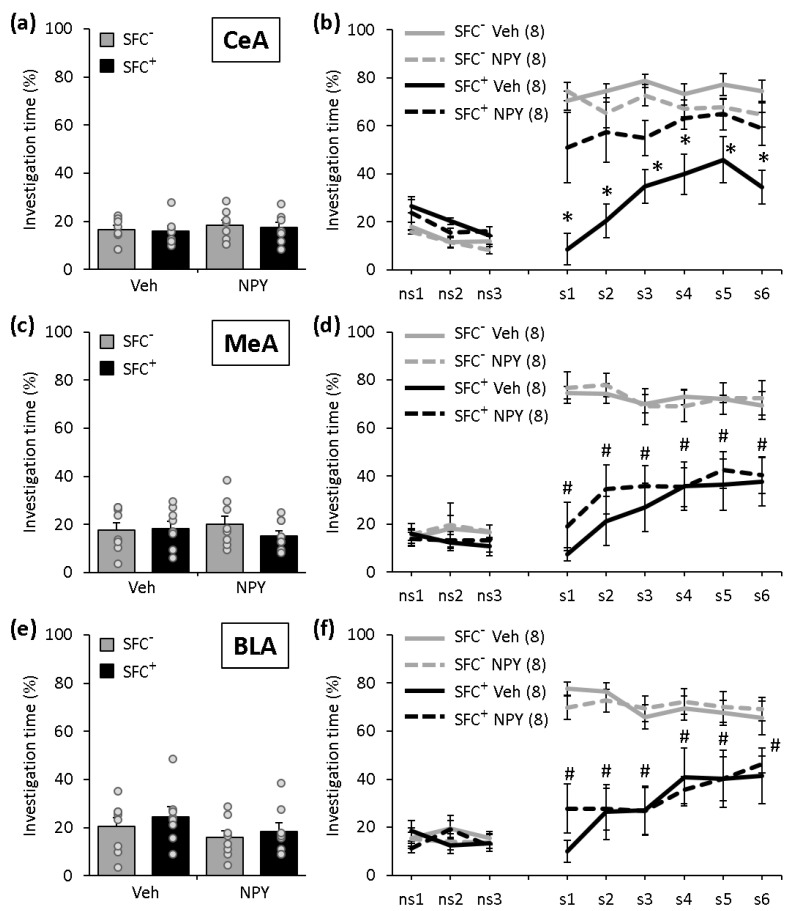
NPY reduces the expression of social fear when administered into the central amygdala (CeA), but not when administered into the medial (MeA) or basolateral amygdala (BLA). (**a**,**c**,**e**) Pre-conditioning investigation of the non-social stimulus (empty cage) during SFC on day 1. (**b**,**d**,**f**) Investigation of the non-social (ns1–ns3) and social (cages with mice; s1–s6) stimuli during social fear extinction on day 2. Unconditioned (SFC^−^) and conditioned (SFC^+^) mice were administered either vehicle (Veh; 0.2 µL/side) or NPY (0.1 nmol/0.2 µL/side) 10 min before social fear extinction. Data represent means ± SEM and numbers in parentheses indicate group sizes. *p* < 0.05 * vs. all groups; # vs. respective SFC^−^ controls.

During SFC on day 1, SFC^+^ and SFC^−^ mice showed similar investigation of the non-social stimulus, reflecting a similar pre-conditioning non-social anxiety (CeA: Figure 3a; F(3,28) = 0.236; *p* = 0.871; MeA: Figure 3c; F(3,28) = 0.429; *p* = 0.734; BLA: Figure 3e; F(3,28) = 1.040; *p* = 0.390). All SFC^+^ mice received a similar number of foot shocks during SFC, reflecting similar levels of distress during conditioning and similar social fear learning (CeA: T(14) = −0.323; *p* = 0.751; MeA: T(14) = −0.851; *p* = 0.409; BLA: T(14) = 1.111; *p* = 0.285). During social fear extinction on day 2 (Figure 3b,d,f), all mice showed a similar non-social investigation, indicating that SFC did not induce an unspecific non-social fear. While all Veh-treated SFC^+^ mice showed social fear, NPY reduced the expression of social fear when administered into the CeA, but not when administered into the MeA or BLA (CeA: Figure 3b; conditioning x treatment effect F(1,28) = 17.210; *p* < 0.001; stimulus x conditioning x treatment effect F(8,224) = 2.769; *p* = 0.006; MeA: Figure 3d; conditioning effect F(1,28) = 50.287; *p* < 0.001; conditioning x treatment effect F(1,28) = 0.237; *p* = 0.630; BLA: Figure 3f; conditioning effect F(1,28) = 43.611; *p* < 0.001; conditioning x treatment effect F(1,28) = 0.113; *p* = 0.739). This indicates that NPY reduces the expression of social fear when administered into the CeA, but not when administered into the MeA or BLA.

### 2.4. NPY Exerts Anxiolytic-Like Effects When Administered into the DH, DLS, CeA and BLA, But Not When Administered into the MeA

**Figure 4:**
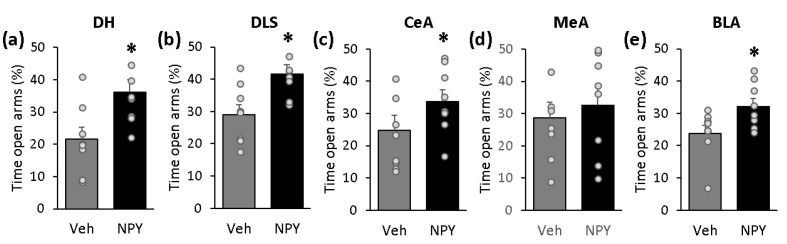
NPY exerts anxiolytic-like effects when administered into the dorsal hippocampus (**a**), dorsolateral septum (**b**), central amygdala (**c**) and basolateral amygdala (**e**), but not when administered into the medial amygdala (**d**). Mice (*n* = 8 per group for each brain region) were administered either vehicle (Veh; 0.2 µL/side) or NPY (0.1 nmol/0.2 µL/side) 10 min before being tested on the elevated plus-maze. Data represent means + SEM. *p* < 0.05 * vs. Veh-treated mice.

We also investigated whether the reduced expression of social fear in NPY-treated SFC^+^ mice might be due to a reduced anxiety-like behavior. By decreasing anxiety-like behavior, NPY might enable SFC^+^ mice to approach the social stimuli faster and, thereby, to express less social fear. To investigate whether NPY alters anxiety-like behavior when administered into the DH, DLS, CeA, MeA and/or BLA, mice were administered either Veh (0.2 µL/side) or NPY (0.1 nmol/0.2 µL/side) into these brain regions 10 min before they were tested on the elevated plus-maze (EPM). NPY exerted anxiolytic-like effects when administered into the DH (Figure 4a; T(14) = −2.699; *p* = 0.017), DLS (Figure 4b; T(14) = −2.845; *p* = 0.013), CeA (Figure 4c; T(14) = −2.202; *p* = 0.045) and BLA (Figure 4e; T(14) = −2.325; *p* = 0.036), as indicated by the increased percentage of time spent on the open arms of the EPM in NPY-treated mice compared with Veh-treated mice. When administered into the MeA, however, NPY did not alter anxiety-like behavior (Figure 4d; T(14) = −0.527; *p* = 0.606). This indicates that NPY exerts anxiolytic-like effects when administered into the DH, DLS, CeA and BLA, but not when administered into the MeA, and suggests that partly distinct neural circuitries mediate the effects of NPY on the expression of social fear and on anxiety-like behavior.

NPY did not alter locomotor activity when administered into any of these brain regions, as indicated by the similar number of entries into the closed arms of the EPM between NPY-treated and Veh-treated mice (DH: T(14) = −0.396; *p* = 0.698; DLS: T(14) = −0.284; *p* = 0.780; CeA: T(14) = −0.715; *p* = 0.486; MeA: T(14) = −0.444; *p* = 0.664; BLA: T(14) = −0.402; *p* = 0.693). This suggests that the anxiolytic-like effects (i.e., the increased time spent on the open arms of the EPM) observed after NPY administration are not due to a general increase in locomotor activity.

## 3. Discussion

The present study demonstrates, for the first time, that NPY reduces the expression of SFC-induced social fear in a brain-region-dependent manner in male mice. In more detail, we could show that when administered into the DLS and CeA, NPY reduced the expression of social fear. In contrast, when administered into the DH, MeA and BLA, NPY did not affect the expression of social fear. We could also show that NPY exerted anxiolytic-like effects when administered into the DH, DLS, CeA and BLA, but not when administered into the MeA. These results suggest that distinct brain regions are recruited to mediate the effects of NPY on the expression of social fear and on anxiety-like behavior.

In previous studies, we have shown that i.c.v.-administered NPY reduces the expression of SFC-induced social fear [34,35]. Here, we extended these findings and localized these effects of NPY in the DLS and CeA. The amygdala is the central component of the fear circuitry and is involved in the perception, learning and expression of fear. Within the amygdala, the CeA constitutes the output relay for the functional consequences of amygdala activation by fearful stimuli, and together with the BLA, coordinates the behavioral and physiological correlates of fear expression [40,41]. Although Fendt et al. [29] have shown that NPY decreased the expression of conditioned fear when administered into the amygdala, a direct involvement of the CeA in the effects of NPY on conditioned fear was not reported previously. The BLA seems to mediate the fear-reducing effects of NPY in a stimulus-specific manner. As such, NPY inhibited the expression of cued fear when administered into the BLA [28], but did not reduce the expression of social fear when administered into the BLA (Figure 3f), suggesting that the BLA mediates the effects of NPY on cued (non-social) fear, but not on social fear. Alternatively, differences in the form of conditioning (e.g., classical conditioning for cued fear versus operant conditioning for social fear) might contribute to the differential role of BLA NPY on fear expression. The MeA was implicated to a lesser extent in fear processes and does not seem to mediate the effects of NPY on conditioned fear, as NPY did not affect the expression of cued fear [28] or the expression of social fear (Figure 3d) when administered into the MeA. The septum and the hippocampus are key components of the behavioral inhibition system regulating emotional behavior and cognitive functions, especially learning and memory, and the septo-hippocampal circuits are important for fear-related behaviors [42,43]. The hippocampus also processes information about the context, i.e., the environment of a fearful situation [43]. The DLS was previously shown to mediate the effects of another neuropeptide, oxytocin, on the expression of social fear [44]. This suggests that multiple neuropeptide systems, including oxytocin and NPY, particularly at the level of the DLS, are regulating the expression of social fear. The DH does not seem to be involved in the effects of NPY on the expression of social fear, which indicates that the processing of context-dependent information related to social fear might not be regulated by NPY.

Similar to its i.c.v. effects [34,35], NPY increased social investigation only in SFC^+^ mice, but not in SFC^−^ mice when administered into the DLS and CeA, suggesting that NPY increases social investigation only in individuals with low or impaired sociability. This resembles the effects of other neuropeptides, such as oxytocin or neuropeptide S, which were also shown to reduce social fear in SFC^+^ mice without further increasing social investigation in SFC^−^ mice [44,45].

We also investigated whether the reduced expression of social fear in NPY-treated SFC^+^ mice might be due to a reduced anxiety-like behavior. By decreasing anxiety-like behavior, NPY might enable SFC^+^ mice to approach the social stimuli faster and, thereby, to express less social fear. Even though we observed anxiolytic-like effects after the administration of NPY both in the DLS and in the CeA, confirming previous findings [17,19], these anxiolytic-like effects are unlikely to completely explain the effects of NPY on the expression of social fear, especially because similar anxiolytic-like effects occurred after administration of NPY into the DH [20] (Figure 4a) and BLA (Figure 4e), regions in which NPY did not reduce the expression of social fear. Interestingly, Sajdyk et al. [24,46] reported anxiolytic-like effects of NPY after administration into the DLS and BLA, but not into the CeA in the social interaction test, a validated test for assessing both anxiety-like behavior [47] and social behavior [48] in rodents. These partly different results might suggest that the neural circuitries mediating the effects of NPY on social behavior and on anxiety-like behavior are different to some extent.

Taken together, we have shown that NPY reduces the expression of SFC-induced social fear when administered into the DLS and CeA, but not when administered into the DH, MeA and BLA. We also showed that NPY exerted anxiolytic-like effects when administered into the DH, DLS, CeA and BLA, but not when administered into the MeA. These results suggest that partly distinct neural circuitries mediate the effects of NPY on the expression of social fear and on anxiety-like behavior.

## 4. Materials and Methods

### 4.1. Animals

CD1 mice (Charles River, Sulzfeld, Germany, 10 weeks of age) were individually housed for 1 week before experiments started and remained single-housed throughout the experiments. Mice were kept under standard laboratory conditions (12:12 light/dark cycle, lights on at 07:00 h, 22 °C, 60% humidity, food and water ad libitum). Experiments were performed during the light phase, between 09:00 and 14:00 h. All efforts were made to minimize animal suffering and to reduce the number of animals used.

### 4.2. Stereotaxic Cannula Implantation

Implantation of the guide cannula (23 G, 8 mm length; Injecta GmbH, Klingenthal, Germany) for bilateral infusions was performed under ketamine-xylazine anesthesia (intraperitoneal injection of 120 mg/kg Ketavet^®^ and 16 mg/kg Rompun^®^, respectively) as previously described [44,49], 1 mm above the DH (from Bregma: AP − 2.0 mm, L ± 1.5 mm, D + 1.4 mm), DLS (AP + 0.3 mm, L ± 0.5 mm, D + 1.6 mm), CeA (AP − 1.2 mm, L ± 2.8 mm, D + 3.8 mm), MeA (AP − 1.1 mm, L ± 2.5 mm, D + 4.5 mm) or BLA (AP − 1.2 mm, L ± 3.1 mm, D + 3.8 mm). After surgery, mice were handled for 5 days before experiments started.

### 4.3. Intracerebral Infusions

Mice received bilateral DH, DLS, CeA, MeA or BLA infusions of either vehicle (Veh; distilled H_2_O; 0.2 µL/side) or porcine NPY (0.1 nmol/0.2 µL/side; PeptaNova, Sandhausen, Germany) via an infusion cannula (25 G, 9 mm length) inserted into the guide cannula and connected via polyethylene tubing to a Hamilton syringe. The infusion system was left in place for 30 s following the infusion to allow diffusion of the solution.

The correct infusion site was verified (Figure S1); accordingly, 1 DH, 1 DLS, 3 CeA, 2 MeA and 2 BLA cannulas were not implanted correctly, and these mice were excluded from the study. NPY dose and timing of administration were selected based on previous studies [26,49].

### 4.4. Social Fear Conditioning (SFC) Paradigm

To induce social fear, mice were conditioned during SFC and social investigation was assessed during social fear extinction as a read-out of social fear.

*SFC.* SFC was performed with a computerized fear conditioning system (TSE System GmbH, Bad Homburg, Germany) as previously described [34,35,44,45,50,51,52]; see [48] for a schematic representation of the SFC paradigm. Mice were placed in the conditioning chamber (45 × 22 × 40 cm) and, after a 30-s habituation period, an empty wire mesh cage (7 × 7 × 6 cm) was placed as a non-social stimulus near one of the short walls. After 3 min, the non-social stimulus was replaced by an identical cage containing an unfamiliar mouse. Unconditioned (SFC^−^) mice were allowed to investigate this social stimulus for 3 min, whereas conditioned (SFC^+^) mice were given a 1-s mild electric foot shock (0.7 mA) each time they investigated, i.e., made direct contact with the social stimulus. Mice received between 1 and 4 foot shocks, with a variable inter-shock interval, depending on when direct social contact was made. The number of foot shocks was assessed as a measure of distress and social fear learning. Mice were returned to their home cage when no further social contact was made for 2 min (average duration of SFC approximately 10 min). All SFC^+^ mice investigated the social stimulus and could be conditioned. The time mice spent investigating the non-social stimulus, as a pre-conditioning measure of non-social anxiety, was analyzed.

*Social fear extinction.* One day after SFC, mice were exposed in their home cage to three non-social stimuli, i.e., empty cages identical to the cage used during SFC, to assess non-social investigation as a parameter of non-social fear. Mice were then exposed to six unfamiliar social stimuli, i.e., mice enclosed in wire mesh cages, to assess social investigation as a parameter of social fear. Each stimulus was placed near a short wall of the home cage and presented for 3 min, with a 3-min inter-exposure interval. The test was recorded and analyzed using JWatcher (V 1.0, Macquarie University, Sydney, Australia and UCLA, Los Angeles, CA, USA). The non-social investigation was defined as a direct sniffing of the empty cage, whereas social investigation was defined as a direct sniffing of the cage and/or of the social stimulus inside the cage.

### 4.5. Elevated Plus-Maze (EPM) Test

To investigate whether NPY alters anxiety-like behavior, mice were tested on the EPM as previously described [46,51]. An increased percentage of time spent on the open arms (150 lx) indicated reduced anxiety-like behavior. The number of entries into the closed arms (30 lx) during the 5-min testing period indicated locomotor activity.

### 4.6. Statistical Analysis

For statistical analysis, SPSS (Version 24, SPSS Inc., Chicago, IL, USA) was used. Data were analyzed by Student’s *t*-test, one-way ANOVA or three-way ANOVA for repeated measures, followed by a Bonferroni’s post-hoc analysis whenever appropriate. Statistical significance was set at *p* < 0.05.

## Supplementary Materials

The following are available online at https://www.mdpi.com/article/10.3390/ijms22073695/s1.
