# Supplementary material for: Brain Region-Dependent Effects of Neuropeptide Y on Conditioned Social Fear and Anxiety-Like Behavior in Male Mice
Source: Int J Mol Sci. 2021 Apr 2;22(7):3695. doi: 10.3390/ijms22073695 (PMC8037261; doi:10.3390/ijms22073695)
Supplement: Supplementary file 1 [file ijms-22-03695-s001.pdf]

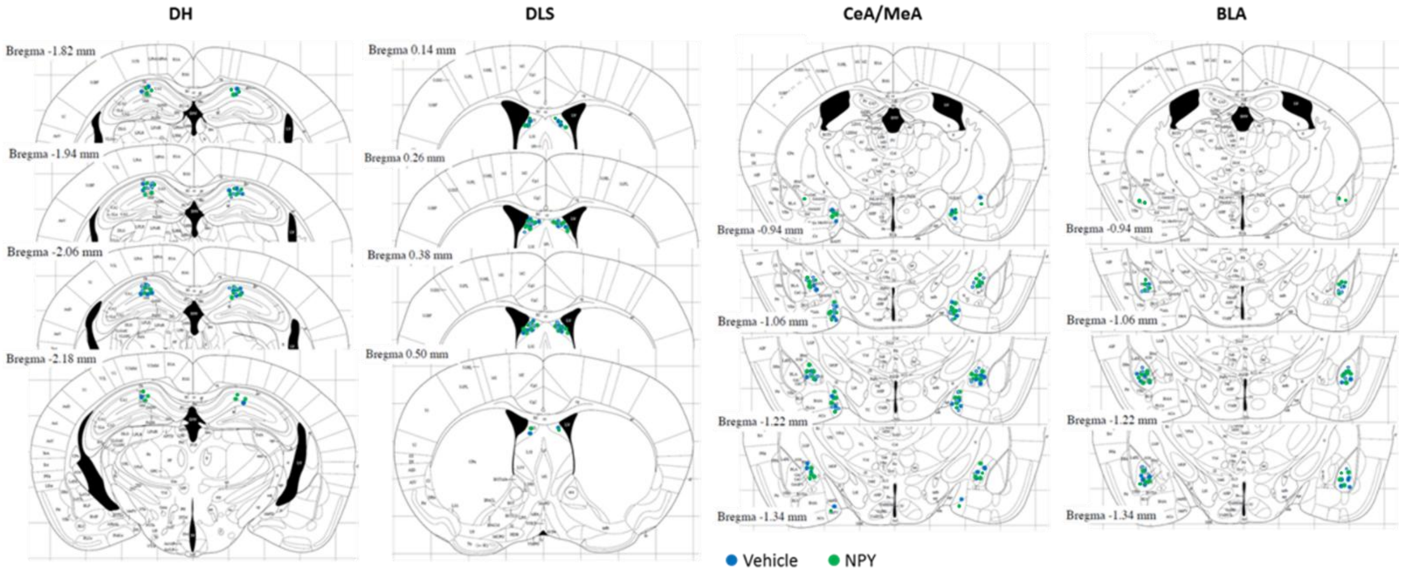

Figure S1. Schematic illustration of coronal sections of the mouse brain showing the infusion sites. DH, dorsal hippocampus; DLS, dorsolateral septum; CeA, central amygdala; MeA, medial amygdala; BLA, basolateral amygdala; NPY, neuropeptide Y.
